# The Repurposing of Non-Peptide Neurokinin-1 Receptor Antagonists as Antitumor Drugs: An Urgent Challenge for Aprepitant

**DOI:** 10.3390/ijms242115936

**Published:** 2023-11-03

**Authors:** Rafael Coveñas, Francisco D. Rodríguez, Prema Robinson, Miguel Muñoz

**Affiliations:** 1 Laboratory of Neuroanatomy of the Peptidergic Systems, Institute of Neurosciences of Castilla y León (INCYL), University of Salamanca, 37007 Salamanca, Spain; covenas@usal.es; 2 Group GIR-BMD (Bases Moleculares del Desarrollo), University of Salamanca, 37007 Salamanca, Spain; lario@usal.es; 3 Department of Biochemistry and Molecular Biology, Faculty of Chemical Sciences, University of Salamanca, 37007 Salamanca, Spain; 4 Department of Infectious Diseases, Infection Control, and Employee Health, MD Anderson Cancer Center, The University of Texas, Houston, TX 77030, USA; 5 Pediatric Intensive Care Unit, Research Laboratory on Neuropeptides (IBIS), Virgen del Rocío University Hospital, 41013 Seville, Spain; mmunoz@cica.es

**Keywords:** NK-1 receptor antagonist, antitumor, anticancer drugs, cancer, Aprepitant, substance P, repurposing

## Abstract

The substance P (SP)/neurokinin-1 receptor (NK-1R) system is involved in cancer progression. NK-1R, activated by SP, promotes tumor cell proliferation and migration, angiogenesis, the Warburg effect, and the prevention of apoptosis. Tumor cells overexpress NK-1R, which influences their viability. A typical specific anticancer strategy using NK-1R antagonists, irrespective of the tumor type, is possible because these antagonists block all the effects mentioned above mediated by SP on cancer cells. This review will update the information regarding using NK-1R antagonists, particularly Aprepitant, as an anticancer drug. Aprepitant shows a broad-spectrum anticancer effect against many tumor types. Aprepitant alone or in combination therapy with radiotherapy or chemotherapy could reduce the sequelae and increase the cure rate and quality of life of patients with cancer. Current data open the door to new cancer research aimed at antitumor therapeutic strategies using Aprepitant. To achieve this goal, reprofiling the antiemetic Aprepitant as an anticancer drug is urgently needed.

## 1. Introduction

Many studies have demonstrated that peptides such as neurotensin [1], angiotensin II [2], calcitonin gene-related peptide [3], somatostatin [4], and hypocretin 1 [5] are involved in cancer development. In recent years, the knowledge of the involvement of the substance P (SP)/neurokinin-1 receptor (NK-1R) system in cancer progression has notably increased [6,7,8,9,10]. It is currently known that tumor cells express receptors for peptides, explicitly emphasizing the overexpression of NK-1R reported in these cells [11]. This finding opens the door to new cancer research avenues, cancer diagnosis, and antitumor therapeutic strategies using NK-1R agonists/antagonists-based cancer therapy, cytotoxic peptide conjugate-based cancer therapy, or peptide-receptor radionuclide therapy [12,13,14,15,16,17,18,19,20]. Radiopharmaceuticals based on non-peptide compounds, such as the drug Aprepitant, a non-peptide NK-1R antagonist used in clinical practice as an antiemetic, have shown great potential in imaging studies, diagnosis, and treatment of tumors that overexpress NK-1R [16].

Based on previous in vitro and in vivo studies, the use of NK-1R antagonists such as Aprepitant is an encouraging anticancer strategy because these antagonists show a broad-spectrum anticancer effect against leukemia, sarcoma, glioma, neuroblastoma, retinoblastoma, osteosarcoma, hepatoblastoma, melanoma, or carcinoma, as a result of their ability to promote apoptosis in cancer cells [6,7,8,9,10,21]. NK-1R antagonists bind to NK-1R expressed in tumor cells and block all the favorable effects mediated by SP on tumor cells, such as proliferation and migration, prevention of apoptosis, and the Warburg effect (glycolytic rate increase) and angiogenesis [22]. Most importantly, novel research strategies must be developed to better understand the in-depth function–structure relationships between SP and NK-1R. These strategies will serve as a tool for designing and synthesizing new, selective, and more effective NK-1R antagonists. Moreover, targeting novel NK-1R-specific sites can lead to the discovery of drugs that can treat the numerous pathologies in which the SP/NK-1R system participates, such as cancer, pain, and depression [23]. This review will provide important information regarding the potential use of NK-1R antagonists as anticancer drugs, particularly Aprepitant in clinical practice as an antitumor drug.

## 2. Involvement of the SP/NK-1R System in Cancer

**Figure 1:**
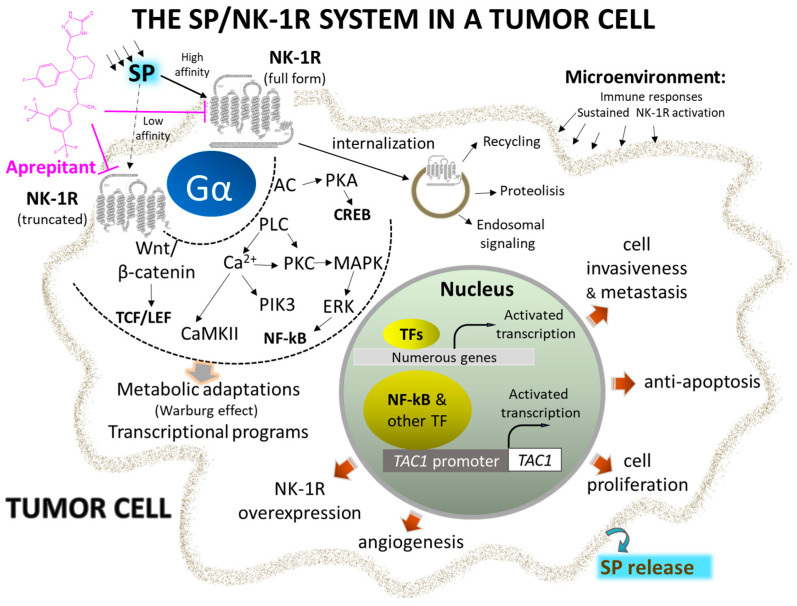
Representative signaling pathways and activated transcriptional programs implicating complete and truncated NK-1R isoforms in the initiation and metastasis of cancer cells. Overexpression of NK-1R and agonist overstimulation induce metabolic changes and transcriptional programs, leading to uncontrolled cell proliferation and migration. Selective and potent antagonists, such as Aprepitant, block the signal and stimulate apoptosis of cancer cells (see text for details). Abbreviations: AC, adenylyl cyclase; CaMKII, calcium-calmodulin kinase II; CREB, cAMP response element; ERK, extracellular-activated kinase; NF-kB, nuclear factor kappa-light-chain-enhancer of activated B cells; NK-1R, neurokinin 1-receptor; PIK3, phosphatidylinositol 3 kinase; PKC, protein kinase C; PLC, phospholipase C; SP, substance P; TAC1, tachykinin precursor 1; TCF/LEF, T cell factor/lymphoid enhancer factor family of transcription factors; Wnt, wingless and Int-1.

The general characteristics of the SP/NK-1R system and the primary data demonstrating its involvement in cancer progression are included in the following thirteen key points [6,7,24,25,26,27,28,29,30,31,32,33,34,35,36] (Figure 1):

1. NK-1R belongs to the rhodopsin-like G protein-coupled receptors family and shows a preferential affinity for SP [37]. This peptide is the natural ligand for NK-1R, so NK-1R is also named the SP receptor [38,39]. The SP high-affinity receptor NK-1R is widely distributed throughout the body and can bind to hemokinin-1, endokinins, and neurokinins. For a recent review focused on the structural dynamics and signaling of NK-1R, see [23].
2. The tachykinin receptor 1 human gene is on chromosome 2 and encodes NK-1R. The pre-protachykinin A human gene on chromosome 7 encodes SP [40]. Tumor cells overexpress the former gene [6,41].
3. The 5’ flanking region of the tachykinin receptor 1 gene contains conserved gene promoter regulatory elements such as the octamer binding protein 2, nuclear factor kappa-light-chain enhancer of activated B cells (NF-κB), activating protein-1, -2, and -4, and cAMP responsive element binding sites. NF-κB favors cell survival, DNA transcription, and cancer progression, blocks apoptosis, and promotes cancer resistance and the synthesis of tumor-associated cytokines (interferon γ, macrophage inflammatory protein-1β, tumor necrosis factor α, interleukins 1β and 6) in tumor cells [42].
4. NK-1R is coupled to Gαs, Gαq, Gαo, Gαi, and Gα_12/13_ proteins; activation of a specific G protein, which differs in the signaling/effector pathways they activate, is controlled by the conformations of NK-1R (each conformation shows a distinct affinity for antagonists and agonists) and type of ligands [43,44,45,46,47,48,49]. Aprepitant promotes conformational changes that interfere with the binding of SP, leading to a long-lasting inhibition of NK-1R [22,50,51,52]. The interaction between SP central and N-terminal regions with the NK-1R extracellular domain is crucial for signaling through Gs. NK-1R antagonists block access to the receptor binding site and hinder G-protein activation [53].
5. SP binds to the extracellular loops of NK-1R [54] and non-peptide NK-1R antagonists between the receptor’s III and VI transmembrane segments; specific amino acid residues (His197, Gln165) control the binding of these antagonists as reported in recent detailed structural studies [48,52,53,55]. The amidated C-terminal of SP is involved in peptide activity since its deamidation suppressed the activity of SP. The C-terminal sequence contains a hydrophobic amino acid residue needed to activate NK-1R [53].
6. Cancer cells express/overexpress NK-1R [11,56,57,58,59,60,61,62,63,64], which is involved in the viability of these cells. It has been suggested that the higher the number of NK-1R, the higher the tumor malignancy, and NK-1R mRNA expression is lower in benign compared to malignant tissues. NK-1R is not involved in the viability of normal cells. Tumor cells express and release SP, but SP is not involved in the viability of cancer cells [65].
7. SP favors the proliferation of tumor cells [66,67,68,69,70,71,72]. The peptide, through NK-1R, promotes the proliferation via mitogen-activated protein kinases (MAPK) signaling and migration of both solid and non-solid cancer cells via the expression of matrix metalloproteinase 9 and exerts an antiapoptotic action by activating protein kinase B (this has been associated with a poor prognosis). SP favors the Warburg effect (glycolytic rate increase), which cancer cells use to maintain their high metabolism rate. SP promotes angiogenesis, leading to neovascularization [73]. Consequently, rapidly multiplying cancer cells use the nutrients and oxygen provided due to SP-induced angiogenesis. SP also increases the expression of NK-1R but not that of the other two tachykinin receptors (NK-2R and NK-3R).
8. NK-1R shows two isoforms: full-length (407 amino acids) and truncated (311 amino acids; C-terminus 96 residues are lost) [74,75]. Isoforms can trigger different intracellular signaling pathways and play different roles in physiological and pathophysiological mechanisms [49]. The full-length form shows a ten-fold higher binding affinity for SP than the truncated form. Cells expressing the full-length form respond to nanomolar concentrations of SP, whereas those cells expressing the truncated isoform need micromolar concentrations of the peptide to elicit a signaling response. The full-length isoform is involved in NK-1R desensitization and internalization, whereas the truncated form partially disrupts signaling pathways but does not affect the SP binding domain. Truncation of the receptor leads to impairment in the receptor being internalized, thus imparting the receptor with (a) resistance to desensitization, (b) weaker interaction with G proteins and protein kinase K and other phosphorylation processes, and (c) delay in Ca^++^ release ultimately resulting in an inhibited response to SP.
9. Tumor cells show a higher level of truncated and a lower level of full-length NK-1R than normal cells. MicroRNA-206 overexpression favors cancer cell proliferation, invasion, and migration via targeting the full-length form, whereas microRNA-22 blocks all these processes by targeting the truncated NK-1R form [67]. Furthermore, NK-1R is the predicted target of the miR-34 family, and the overexpression of microRNA-34b/c-5p has been shown to suppress cancer cell proliferation and promote apoptosis via NK-1R suppression.
10. The truncated NK-1R isoform is involved in malignancy, tumor cell growth, metastasis, and apoptosis blockade [76]. In contrast, the full-length expression, inversely associated with invasion and metastasis, decreases cancer cell proliferation and attenuates apoptotic signals. Truncated NK-1 expression is positively regulated via Smad4 by tumor growth factor β and blocked with NK-1R antagonists (Aprepitant). SP promotes the activation of NF-κB, which upregulates the truncated form, induces a slight increase in the full-length isoform, and favors resistance to some chemotherapeutic agents and Aprepitant. This observation is vital since tumors showing overexpression of the NF-κB pathway may need to be treated with a higher dose of the NK-1R antagonist for mediating anticancer effects.
11. SP is synthesized and released by cancer and immune cells, and it is released from nerve terminals and circulates in the bloodstream [62,77,78,79]. SP-immunoreactive fibers have been associated with tumor differentiation status. SP acts through autocrine, paracrine, neuroendocrine, and endocrine (from the tumor mass) mechanisms. SP and NK-1R have also been observed in the nuclei of cancer cells; their physiological significance is currently unknown.
12. Following the interaction of SP with NK-1R, important downstream events are activated, such as diacylglycerol synthesis with the resultant activation of protein kinase C and promotion of the influx of extracellular Ca^++^ via calcium channels. Downstream events, such as inositol triphosphate production, promote Ca^++^ release from the endoplasmic reticulum into the cytoplasm. High calcium levels enhance proliferative and pro-survival pathways such as MAPK and extracellular signal-regulated kinases (ERK). Furthermore, calcium governs other essential processes such as cell death, migration, communication, and immune activation [80]. NK-1R antagonists promote rapid endoplasmic reticulum/mitochondria Ca^++^ overload and accumulation of reactive oxygen species, causing apoptosis. In conclusion, since the processes mentioned above can be deregulated and exploited by cancer cells that overexpress NK-1R, this receptor can serve as a suitable therapeutic target in cancer [50,81,82].
13. Although both normal and cancer cells produce SP, lower levels of SP are detected in normal cells compared with cancer cells. More serum SP and NK-1R concentrations were found in cancer patients than healthy individuals. NK-1R overexpression has been associated with larger tumor size, higher metastatic and invasion potential tumor-node metastasis, advanced cancer stages, and poor prognosis. Thus, NK-1R overexpression and high serum SP levels could be used as predictive biomarkers for increased risk of developing cancer and cancer prognosis [57,59].

The survival of tumor cells can be decreased and blocked by applying several strategies. Apoptotic mechanisms were observed in tumor cells when treated with antibodies against SP when the blockade of the SP signal was performed using NK-1R antagonists or when the silencing of the NK-1 expression was carried out [65,83]. In addition, the synthesis of cell cycle proteins halted when tumor cells did not receive the SP stimulus. The previous studies show the crucial role that the SP stimulus mediated by NK-1R exerts on tumor cells since the peptide promotes beneficial effects for the survival of cancer cells, such as proliferation/migration, the Warburg effect, and antiapoptotic action.

The SP/NK-1R system can be manipulated for the following therapeutic benefits: (a) tumor cells treated with antibodies against SP, with NK-1R antagonists or NK-1R expression inhibition, promoted apoptotic mechanisms [65,83,84], and (b) halted the synthesis of cell cycle proteins occurring when tumor cells do not receive the SP stimulus. It seems that tumor cells overexpress NK-1R to ensure the SP signaling and utilize this stimulus to mediate tumorigenic effects. Still, at the same time, cancer cells are impacted by the signal mediated by SP because NK-1R overexpression renders cancer cells extremely dependent on the SP stimulus. If tumor cells do not receive this stimulus, apoptotic mechanisms turn on in these cells. Importantly, it seems that this does not occur in normal cells. This dependence on the SP stimulus is essential from a therapeutic point of view because NK-1R represents a specific therapeutic target for cancer treatment, and it could explain why NK-1R is involved in the viability of tumor cells but not in the viability of normal cells [65]. Accordingly, a question arises: is NK-1R the Achilles’ heel of cancer cells? Many data support this hypothesis.

## 3. NK-1R Antagonists as Anticancer Drugs

**Figure 2:**
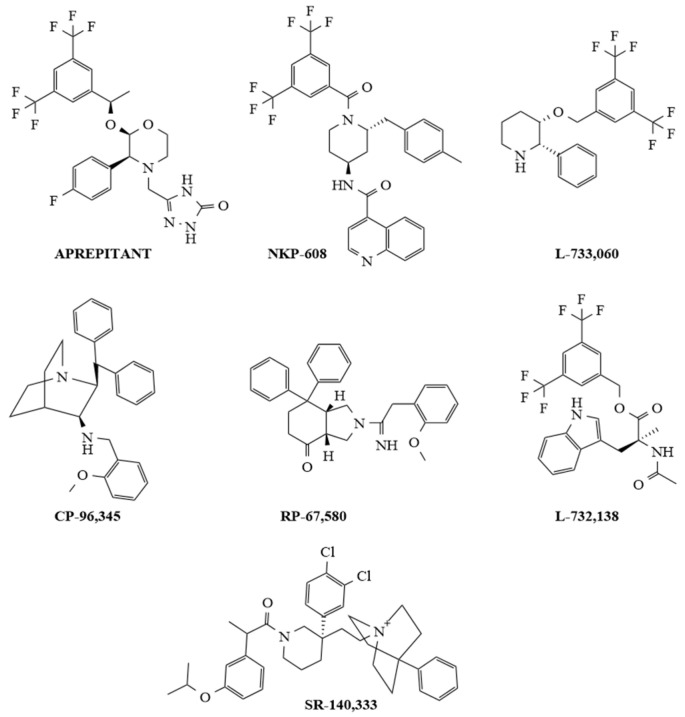
Chemical structures of representative selective non-peptide NK-1R antagonists.

Non-peptide NK-1R antagonists are lipid-soluble compounds that show a different chemical composition (benzyl ether piperidines, benzylamine/benzyl ether quinuclidine, benzyl amino piperidines, steroids, tryptophan derivatives, perhydro isoindolines) (Figure 2) but have a similar affinity for NK-1R [52,85].

SP promotes blood–brain barrier breaching by tumor cells, and most importantly, non-peptide NK-1R antagonists easily cross the blood–brain barrier, and peptidases do not degrade these antagonists. Additionally, non-peptide NK-1R antagonists decrease the toxicity of cytostatics and the permeability of tumor cells across this barrier (preventing brain metastasis); in general, they are safe and well tolerated [6,81,86,87]. Many in vitro and in vivo studies have demonstrated that NK-1R antagonists (Aprepitant, L-732,138, L-733,060, CP-96,345, SR-140,333, NKP-608, RP-67,580) are broad-spectrum anticancer agents that act in a concentration-dependent manner [22,88,89,90,91,92,93,94,95,96,97,98,99,100,101,102,103,104]; one of the primary mechanisms by which these antagonists induce an antitumor effect is by causing the death of many different cancer cell types by apoptosis (see Table 1 and Figure 2).

Testing the anticancer action of NK-1R antagonists in other tumors, such as oral squamous cell carcinoma and uterine leiomyomata, is worthwhile since all these tumor types express NK-1R (Table 1) [11,129]. It is important to note that the antiemetic drug Aprepitant is the NK-1R antagonist exerting an anticancer action (apoptosis) against as many as 21 different tumor types (Table 1); this is pivotal for its repurposing as an anticancer drug. As stated above, SP impairs the blood–brain barrier, thus facilitating the invasion of cancer cells into the central nervous system; NK-1R antagonists have been shown to prevent this invasion by cancer cells [86]. The SP/NK-1R interaction pathway induces the migration of tumor cells via Rho-associated protein kinase (ROCK)-mediated signaling, leading to the upregulation of expression of matrix metalloproteinase 2, which degrades extracellular matrix proteins [41,130,131]. NK-1R antagonists are known to prevent tumor cell proliferation, invasion, and metastasis via suppression of the Wnt/β-catenin signaling pathway, and it has been suggested that, before and after cancer surgical procedures, Aprepitant could be administered to prevent metastasis and recurrence [132,133]. Cancer cells showing high levels of the truncated NK-1R form are highly responsive to NK-1R antagonists, and this could be important for the specific and safe use of NK-1R antagonists since tumor cells express more of this form than normal cells [103]

Moreover, NK-1R agonists decreased the expression of Dickkopf 1 (a Wnt inhibitor) and augmented β-catenin and glycogen synthase kinase-3β expressions [134]. The NK-1R antagonist Aprepitant favors caspase-dependent apoptotic mechanisms. It alters the expression of genes involved in cell survival and drug resistance by blocking the phosphatidylinositol 3-kinase (PI3K)/protein kinase B (Akt) signaling cascade. It increases the cleavage of the poly (ADP-ribose) polymerase, an enzyme that repairs DNA damage [95,97,135]. Aprepitant shows mild/moderate side effects: the most common are constipation, diarrhea, headache, hiccups, fatigue, and anorexia; however, other low-incidence side effects (<1%) such as euphoria, disorientation, cognitive disorders, candidiasis, acid reflux, epigastric discomfort, and lethargy have also been reported [81,136,137]. Aprepitant is currently used as an antiemetic but also exerts an antipruritic action in cutaneous lymphoma and acts as a cough suppressant in patients with lung cancer [6,138,139]. Because Aprepitant is poorly water-soluble, strategies (e.g., nanoparticle formulation) must be developed to increase its solubility, dissolution, and efficacy [6]. Finally, a liquid chromatography–tandem mass spectrometry method has recently been developed to quantify the free and total Aprepitant and its active N-dealkylated metabolites in human plasma, allowing accurate measurement in pharmacokinetic studies [140]. Table 1 highlights the NK-1R antagonists exerting an antitumor action (favoring apoptosis) against many different tumors, supporting the use of NK-1R antagonists as antitumor drugs, especially Aprepitant. It is important to remark that no clinical trials have focused on the antitumor action of NK-1R antagonists. One of the main goals of this review is to provide data that support the development of clinical trials using Aprepitant as an antitumor drug, but before the repurposing of antiemetic Aprepitant is needed. The only NK-1R antagonist in Table 1 currently used in clinical practice is the antiemetic Aprepitant. Consequently, it is the best candidate NK-1R antagonist to be reprofiled as an antitumor drug.

## 4. Aprepitant Repurposing

Serious side effects, some of them mediated by the SP/NK-1R system, appear when patients with cancer are treated with chemotherapy, such as neurotoxicity, nephrotoxicity, hepatotoxicity, cardiotoxicity, neutropenic enterocolitis, and neutropenic fever [50]. Moreover, radiotherapy/chemotherapy induces inflammatory processes due to the release of SP from nerve terminals; these processes are attenuated with NK-1R antagonists [50]. SP is an ardent chemoattractant for immune cells (monocytes). It enhances the inflammatory processes by releasing pro-inflammatory mediators from these cells, which promote lymphocyte recruitment and proliferation [78]. In the mucosal barrier, the neurogenic inflammation mediated by SP could promote a systemic disease process exacerbated by neutropenia induced as after-effects of radiation or cytostatics; NK-1R antagonists can inhibit these processes by blocking the triggering of the inflammatory cascade [141]. However, chemotherapy is the most used anticancer treatment despite its severe side effects and drug resistance [6,142]. SP enhances malignancy and resistance in breast cancer cells by the transmodulation of epidermal growth factor receptor (EGFR) and epidermal growth factor receptor 2 (HER2) [143]. Many studies have demonstrated that NK-1R antagonists counteract the side effects of chemotherapy and radiotherapy and that SP/NK-1R mediate cancer chemoresistance through Notch 1, Raf/MEK/ERK and PI3K/Akt/mTOR and MAPK signaling pathways [7,9,144]. These pathways promote cell proliferation, regulate cell metabolism, block apoptosis, and are crucial in cancer chemoresistance [145,146]. Therefore, the SP/NK-1R system is a potential target to increase the response to anticancer immunotherapies [7]. In combination with radiotherapy or chemotherapy, aprepitant induced radiosensitization or chemosensitization, i.e., increased anticancer action mediated by radiotherapy or chemotherapy.

Moreover, normal cells such as fibroblasts and human embryonic kidney 293 cells were protected from cytostatic drugs when NK-1R antagonists were administered before cytostatics [6,88,147,148,149]. The IC_50_ of Aprepitant for acute myeloid leukemia cells is ten-fold lower than that for lymphocytes, thus showing the drug to be very safe [150]. Aprepitant also decreased the harsh side effects mediated by cisplatin (nephrotoxicity, neurotoxicity, hepatotoxicity), increased the anticancer effect of cisplatin, and overcame resistance to cytostatics through the blockade of the ERK-c-myc signaling [6,9]. Recent studies have been published on the beneficial antitumor actions of the combination therapy of Aprepitant with radiotherapy or chemotherapy [6]. The promising effects of the combination therapy of Aprepitant with cisplatin, doxorubicin, 5-fluorouracil, cytosine arabinoside, temozolomide, or ritonavir have been established, such as chemosensitization and decrease in the side effects mediated by the different chemotherapeutic drugs [6]. For example, Aprepitant increased and decreased the antitumor effect (chemosensitization) and the toxicity of cytosine arabinoside in tumor cells; these effects were also observed in cancer cells when Aprepitant was co-administered with cisplatin. Aprepitant counteracted the cardiotoxicity and chemoresistance promoted by doxorubicin and the side effects mediated by the antitumor drug erlotinib, an inhibitor of the epidermal growth factor receptor tyrosine kinase [6]. In addition, Aprepitant increased the antitumor activity exerted by ritonavir and temozolomide. Thus, Aprepitant increases the therapeutic effect of various chemotherapeutic agents and, at the same time, decreases the non-desirable severe side effects mediated by them. Aprepitant and cytostatics (doxorubicin, cisplatin) showed a synergic antitumor action against hepatoblastoma cells, and a synergic effect was also reported in osteosarcoma cells when the NK-1R antagonist L-733,060 was co-administered with cytostatics such as cisplatin, ifosfamide, mitomycin, adriamycin; however, this effect was not reported in non-malignant human embryonic kidney 293 cells [88,151].

A case report has been published in which the combination therapy of palliative radiotherapy and compassionate use of Aprepitant (1140 mg/day for 45 days) was reported. This therapy was applied in a patient with lung cancer; six months after treatment, the tumor mass (8 × 7 cm) disappeared, and no side effects were observed [152]. This finding is vital since, compared with the low doses administered in clinical practice as antiemetic (only three days: 125 mg, 80 mg, and 80 mg), the previous study demonstrates that high antitumor doses of Aprepitant administered during a long time are safe and well-tolerated and, in addition, this coincides with earlier studies in which higher doses of Aprepitant (375 mg/day for two weeks; 80 mg/day for seven months) were safe and well tolerated [81,152]. The correct antitumor dose of Aprepitant relates to the total number of NK-1R, particularly the total number of the truncated NK-1R form, and the size of the targeted tumor (larger size, higher dose) [141]. This is important since the different expression patterns of NK-1R isoforms could be used to select patients with cancer who could be treated in a personalized manner with NK-1R antagonists alone or as adjuvant therapy [8]. An NK-1R occupancy close to 100% is required for a good efficacy of NK-1R antagonists; this is another crucial point for NK-1R antagonists to exert an anticancer action [153,154]. Aprepitant displays some harmful effects: it increases the plasma levels of corticosteroids and chemotherapeutic agents and the risk of developing chemotherapy-induced peripheral neuropathy. Antitumor doses of Aprepitant have been reported in experimental animals [50,81]; however, these doses were much higher (125–2000 mg/kg/day) compared to the antitumor doses of Aprepitant suggested to be administered in humans: 20–40 mg/kg/day [132].

Moreover, the risk of febrile neutropenia promoted by Aprepitant in pediatric bone cancer patients has been reported [155]. These harmful actions mediated by Aprepitant must be better studied and defined. A study has reported that Aprepitant exerts an antiproliferative activity against melanoma, lung cancer, and urinary bladder carcinoma cell lines; however, contrary to most of the published works, the authors reported that Aprepitant was not selective, and it affected normal and cancer cell lines to a similar degree [100]. The unknown discrepancy regarding the antiproliferative action of Aprepitant against normal cells must be elucidated in future studies. The authors also indicated that the tumor cell lines tested in their study were more resistant to Aprepitant than those cancer cell lines tested previously by other authors. These studies need to be further tested and clarified.

Chemotherapy can promote chemoresistance, and most deaths (90%) occurring in individuals treated with new antitumor treatments or chemotherapy are due to drug resistance [156]. The repurposing of Aprepitant has recently been suggested to overcome this drug resistance, and the use of Aprepitant alone or in combination therapy with chemotherapy has also been indicated for treating rhabdoid tumors [7,90]. The administration of Aprepitant alone showed a lower antitumor action than when the combination therapy of Aprepitant and chemotherapy was applied [157]. The data confirm that the antiemetic Aprepitant is an excellent anticancer drug and that its repurposing is needed. Phases I and II must be compellingly developed to ascertain the efficacy, safety, tolerability, drug–drug interactions, administration time, and its highest safe dose to exert the maximal anticancer effect of Aprepitant. According to that reported above, the antitumor action exerted by Aprepitant has recently been published against osteosarcoma, esophageal cancer, glioblastoma, and gallbladder cancer [158,159,160]. These data support the use of the antiemetic Aprepitant as an antitumor drug.

## 5. Conclusions

Peptide/receptor genes are linked with the induction of different types of cancer [161]. New anticancer strategies could be focused on drugs targeting tumor-specific molecular derangements; one of these derangements is the overexpression of NK-1R, which is involved in the viability of cancer cells. These findings support using NK-1R as an antagonist capable of specifically targeting cancer cells. Because tumor cells express NK-1R, a common specific anticancer strategy using NK-1R antagonists is possible, irrespective of the tumor type. These antagonists, alone or in combination therapy with radiotherapy/chemotherapy, could reduce the sequelae and increase the cure rate and quality of life of patients with cancer. NK-1R antagonists attenuate the severe side effects promoted by chemotherapy/radiotherapy [85,162,163]. According to previously published works, we suggest administering Aprepitant (20 mg/kg/day) during radiotherapy or in addition to the chemotherapy protocol (20 mg/kg/day, the first week in each cycle). This combination therapy could be a universal and novel antitumor strategy exerting a dual action: a synergic anticancer action and protection from the severe side effects of chemotherapy and radiotherapy. In summary, NK-1R is an anticancer therapeutic target that could improve both the diagnosis and treatment of cancers [164,165]. The currently available data support the reprofiling of the antiemetic Aprepitant as an anticancer drug. Pari passu, the synthesis of new NK-1R ligands may render anticancer agents effective [166,167]. Also, targeting downstream modulators of NK-1R activity to fight cancer cells, as it is the case of long non-coding RNA PVT1, should be considered [168].

## Figures and Tables

**Table 1:** The main non-peptide NK-1R antagonists (aprepitant, L-732,138, L-733,060) and other antagonists studied showed the same antitumor effect (apoptosis) against different tumors. In all cases, including other antagonists, apoptosis was observed in in vitro experiments. +: apoptosis. The asterisk (*) indicates that in vivo experiments were also performed. ↓: tumor volume decrease. ns: not inspected.

Cancer	Aprepitant	L-732,138	L-733,060	Others	References
Acute lymphoblastic leukemia	+	+	+		[51,105,106]
Acute myeloid leukemia	+	+	+	CP-96,345 (+)	[104,105]
Breast cancer	+	+	+	CP-96,345 (+); RP-67,580 (+); SR-140,333 (+)	[102,103,107]
Cervical cancer	+	ns	ns		[89,108]
Chronic myeloid leukemia	+	ns	+		[104]
Colangiocarcinoma *	ns	ns	+↓		[109]
Colorectal carcinoma *	+,↓	+	+	NKP-608 (+); SR-140,333 (+)	[96,97,110,111]
Esophageal carcinoma	+	ns	ns		[95,99,112]
Gallbladder cancer *	ns	ns	ns	L-703,606 (+, ↓)	[70]
Gastric carcinoma	+	+	+		[22]
Glioblastoma multiforme	+	+	+		[15,64,113,114]
Head and neck cancer	ns	ns	ns	L-703,606 (+)	[98]
Hepatoblastoma *	+, ↓	+	+		[115,116,117]
Larynx carcinoma	+	+	+		[118]
Lung cancer	+	+	+		[94]
Melanoma	+	+	+		[119,120,121]
Neuroblastoma	+	+	+		[122,123,124,125]
Oral squamous cell carcinoma	ns	ns	ns		
Osteosarcoma *	+	+	+		[88,91]
Ovarian cancer	+	ns	ns		[92,126]
Pancreatic carcinoma	+	+	+		[127,128]
Prostate cancer *	+, ↓	ns	ns		[93,120]
Retinoblastoma	+	+	+		[22]
Rhabdoid tumors	+	ns	ns		[90]
Urinary bladder carcinoma	+	ns	ns		[100]
Uterine leiomyomata	ns	ns	ns		

## References

[1] Sánchez M.L., Coveñas R. (2022). The Neurotensinergic System: A Target for Cancer Treatment. Curr. Med. Chem..

[2] Gu L., Zhu Y., Lee M., Nguyen A., Ryujin N.T., Huang J.Y., Pandit S.K., Chamseddine S., Xiao L., Mohamed Y.I. (2023). Angiotensin II receptor inhibition ameliorates liver fibrosis and enhances hepatocellular carcinoma infiltration by effector T cells. Proc. Natl. Acad. Sci. USA.

[3] Sánchez M.L., Rodríguez F.D., Coveñas R. (2023). Peptidergic Systems and Cancer: Focus on Tachykinin and Calcitonin/Calcitonin Gene-Related Peptide Families. Cancers.

[4] Oliveira H.A., Somvanshi R.K., Kumar U. (2023). Comparative changes in breast cancer cell proliferation and signalling following somatostatin and cannabidiol treatment. Biochem. Biophys. Res. Commun..

[5] Huan R., Yue J., Lan J., Wang J., Cheng Y., Zhang J., Tan Y. (2023). Hypocretin-1 suppresses malignant progression of glioblastoma cells through Notch1 signaling pathway. Brain Res. Bull..

[6] Robinson P., Coveñas R., Muñoz M. (2023). Combination Therapy of Chemotherapy or Radiotherapy and the Neurokinin-1 Receptor Antagonist Aprepitant: A New Antitumor Strategy?. Curr. Med. Chem..

[7] García-Aranda M., Téllez T., McKenna L., Redondo M. (2022). Neurokinin-1 Receptor (NK-1R) Antagonists as a New Strategy to Overcome Cancer Resistance. Cancers.

[8] Beirith I., Renz B.W., Mudusetti S., Ring N.S., Kolorz J., Koch D., Bazhin A.V., Berger M., Wang J., Angele M.K. (2021). Identification of the Neurokinin-1 Receptor as Targetable Stratification Factor for Drug Repurposing in Pancreatic Cancer. Cancers.

[9] Rodriguez E., Pei G., Kim S.T., German A., Robinson P. (2021). Substance P Antagonism as a Novel Therapeutic Option to Enhance Efficacy of Cisplatin in Triple Negative Breast Cancer and Protect PC12 Cells against Cisplatin-Induced Oxidative Stress and Apoptosis. Cancers.

[10] González-Moles M.Á., Ramos-García P., Esteban F. (2021). Significance of the Overexpression of Substance P and Its Receptor NK-1R in Head and Neck Carcinogenesis: A Systematic Review and Meta-Analysis. Cancers.

[11] Mehboob R., Kurdi M., Baeesa S., Fawzy Halawa T., Tanvir I., Maghrabi Y., Hakamy S., Saeedi R., Moshref R., Nasief H. (2022). Immunolocalization of neurokinin 1 receptor in WHO grade 4 astrocytomas, oral squamous cell and urothelial carcinoma. Folia Neuropathol..

[12] Wu Y., Berisha A., Borniger J.C. (2022). Neuropeptides in Cancer: Friend and Foe?. Adv. Biol..

[13] Moody T.W., Czerwinski G., Tarasova N.I., Moody D.L., Michejda C.J. (2004). The development of VIP-ellipticine conjugates. Regul. Pept..

[14] Reubi J.C. (2003). Peptide receptors as molecular targets for cancer diagnosis and therapy. Endocr. Rev..

[15] Matalińska J., Kosińska K., Halik P.K., Koźmiński P., Lipiński P.F.J., Gniazdowska E., Misicka A. (2022). Novel NK1R-Targeted (68)Ga-/(177)Lu-Radioconjugates with Potential Application against Glioblastoma Multiforme: Preliminary Exploration of Structure-Activity Relationships. Int. J. Mol. Sci..

[16] Halik P.K., Lipiński P.F.J., Matalińska J., Koźmiński P., Misicka A., Gniazdowska E. (2020). Radiochemical Synthesis and Evaluation of Novel Radioconjugates of Neurokinin 1 Receptor Antagonist Aprepitant Dedicated for NK1R-Positive Tumors. Molecules.

[17] Halik P.K., Koźmiński P., Matalińska J., Lipiński P.F.J., Misicka A., Gniazdowska E. (2022). In Vitro Biological Evaluation of Aprepitant Based (177)Lu-Radioconjugates. Pharmaceutics.

[18] Królicki L., Kunikowska J., Bruchertseifer F., Koziara H., Królicki B., Jakuciński M., Pawlak D., Rola R., Morgenstern A., Rosiak E. (2020). (225)Ac- and (213)Bi-Substance P Analogues for Glioma Therapy. Semin. Nucl. Med..

[19] Mander K., Harford-Wright E., Lewis K.M., Vink R. (2014). Advancing drug therapy for brain tumours: A current review of the pro-inflammatory peptide Substance P and its antagonists as anti-cancer agents. Recent. Pat. CNS Drug Discov..

[20] Song J., Huang S., Ma P., Zhang B., Jia B., Zhang W. (2020). Improving NK1R-targeted gene delivery of stearyl-antimicrobial peptide CAMEL by conjugating it with substance P. Bioorg. Med. Chem. Lett..

[21] Ding G., Wang T., Han Z., Tian L., Cheng Q., Luo L., Zhao B., Wang C., Feng S., Wang L. (2021). Substance P containing peptide gene delivery vectors for specifically transfecting glioma cells mediated by a neurokinin-1 receptor. J. Mater. Chem. B.

[22] Muñoz M., Rosso M. (2010). The NK-1 receptor antagonist aprepitant as a broad spectrum antitumor drug. Investig. New Drugs.

[23] Rodríguez F.D., Coveñas R. (2022). The Neurokinin-1 Receptor: Structure Dynamics and Signaling. Receptors.

[24] Lai J., Lai S., Tuluc F., Tansky M.F., Kilpatrick L.E., Leeman S.E., Douglas S.D. (2008). Differences in the length of the carboxyl terminus mediate functional properties of neurokinin-1 receptor. Proc. Natl. Acad. Sci. USA.

[25] Koon H., Zhao D., Zhan Y., Moyer M.P., Pothoulakis C. (2007). Substance P mediates antiapoptotic responses in human colonocytes by Akt activation. Proc. Natl. Acad. Sci. USA.

[26] Fulenwider H.D., Smith B.M., Nichenko A.S., Carpenter J.M., Nennig S.E., Cheng K., Rice K.C., Schank J.R. (2018). Cellular and behavioral effects of lipopolysaccharide treatment are dependent upon neurokinin-1 receptor activation. J. Neuroinflamm..

[27] Walczak-Drzewiecka A., Ratajewski M., Wagner W., Dastych J. (2008). HIF-1alpha is up-regulated in activated mast cells by a process that involves calcineurin and NFAT. J. Immunol..

[28] Werge T. (2007). The tachykinin tale: Molecular recognition in a historical perspective. J. Mol. Recognit..

[29] Douglas S.D., Leeman S.E. (2011). Neurokinin-1 receptor: Functional significance in the immune system in reference to selected infections and inflammation. Ann. N. Y. Acad. Sci..

[30] Nässel D.R., Zandawala M., Kawada T., Satake H. (2019). Tachykinins: Neuropeptides That Are Ancient, Diverse, Widespread and Functionally Pleiotropic. Front. Neurosci..

[31] Dam T.V., Escher E., Quirion R. (1988). Evidence for the existence of three classes of neurokinin receptors in brain. Differential ontogeny of neurokinin-1, neurokinin-2 and neurokinin-3 binding sites in rat cerebral cortex. Brain Res..

[32] Tuluc F., Lai J.P., Kilpatrick L.E., Evans D.L., Douglas S.D. (2009). Neurokinin 1 receptor isoforms and the control of innate immunity. Trends Immunol..

[33] Ballesteros J.A., Weinstein H. (1995). [19] Integrated methods for the construction of three-dimensional models and computational probing of structure-function relations in G protein-coupled receptors. Methods Neurosci..

[34] (2023). UniProt Database. https://www.uniprot.org/.

[35] Bootman M.D., Chehab T., Bultynck G., Parys J.B., Rietdorf K. (2018). The regulation of autophagy by calcium signals: Do we have a consensus?. Cell Calcium.

[36] Javid H., Mohammadi F., Zahiri E., Hashemy S.I. (2019). The emerging role of substance P/neurokinin-1 receptor signaling pathways in growth and development of tumor cells. J. Physiol. Biochem..

[37] IUPHAR/BPS Guide to Pharmacology Database (2022). Tachykinin Receptors (Version 2019.4). http://journals.ed.ac.uk/gtopdb-cite/article/view/3214/4264.

[38] Muñoz M., Coveñas R., Huhtaniemi I., Martini L. (2019). Substance P. Encyclopedia of Endocrine Diseases.

[39] Goldsmith L.E., Kwatra M.M., Lennarz W.J., Lane M.D. (2013). Tachykinin/substance P/neurokinin-1 receptors. Encyclopedia of Biological Chemistry.

[40] Muñoz M., Coveñas R., Offermanns S., Rosenthal W. (2021). Neurokinin/Tachykin receptors. Encyclopedia of Molecular Pharmacology.

[41] Li X., Ma G., Ma Q., Li W., Liu J., Han L., Duan W., Xu Q., Liu H., Wang Z. (2013). Neurotransmitter substance P mediates pancreatic cancer perineural invasion via NK-1R in cancer cells. Mol. Cancer Res..

[42] Bentires-Alj M., Barbu V., Fillet M., Chariot A., Relic B., Jacobs N., Gielen J., Merville M., Bours V. (2003). NF-kappaB transcription factor induces drug resistance through MDR1 expression in cancer cells. Oncogene.

[43] Roush E.D., Kwatra M.M. (1998). Human substance P receptor expressed in Chinese hamster ovary cells directly activates G(alpha q/11), G(alpha s), G(alpha o). FEBS Lett..

[44] Mitsuhashi M., Ohashi Y., Shichijo S., Christian C., Sudduth-Klinger J., Harrowe G., Payan D.G. (1992). Multiple intracellular signaling pathways of the neuropeptide substance P receptor. J. Neurosci. Res..

[45] Sagan S., Chassaing G., Pradier L., Lavielle S. (1996). Tachykinin peptides affect differently the second messenger pathways after binding to CHO-expressed human NK-1 receptors. J. Pharmacol. Exp. Ther..

[46] Takeda Y., Blount P., Sachais B.S., Hershey A.D., Raddatz R., Krause J.E. (1992). Ligand binding kinetics of substance P and neurokinin A receptors stably expressed in Chinese hamster ovary cells and evidence for differential stimulation of inositol 1,4,5-trisphosphate and cyclic AMP second messenger responses. J. Neurochem..

[47] Fong T.M., Anderson S.A., Yu H., Huang R.R., Strader C.D. (1992). Differential activation of intracellular effector by two isoforms of human neurokinin-1 receptor. Mol. Pharmacol..

[48] Thom C., Ehrenmann J., Vacca S., Waltenspühl Y., Schöppe J., Medalia O., Plückthun A. (2021). Structures of neurokinin 1 receptor in complex with G(q) and G(s) proteins reveal substance P binding mode and unique activation features. Sci. Adv..

[49] Steinhoff M.S., von Mentzer B., Geppetti P., Pothoulakis C., Bunnett N.W. (2014). Tachykinins and their receptors: Contributions to physiological control and the mechanisms of disease. Physiol. Rev..

[50] Muñoz M., Coveñas R. (2020). The Neurokinin-1 Receptor Antagonist Aprepitant: An Intelligent Bullet against Cancer?. Cancers.

[51] Muñoz M., Coveñas R. (2020). The Neurokinin-1 Receptor Antagonist Aprepitant, a New Drug for the Treatment of Hematological Malignancies: Focus on Acute Myeloid Leukemia. J. Clin. Med..

[52] Schöppe J., Ehrenmann J., Klenk C., Rucktooa P., Schütz M., Doré A.S., Plückthun A. (2019). Crystal structures of the human neurokinin 1 receptor in complex with clinically used antagonists. Nat. Commun..

[53] Harris J.A., Faust B., Gondin A.B., Dämgen M.A., Suomivuori C., Veldhuis N.A., Cheng Y., Dror R.O., Thal D.M., Manglik A. (2022). Selective G protein signaling driven by substance P-neurokinin receptor dynamics. Nat. Chem. Biol..

[54] Bremer A.A., Leeman S.E., Boyd N.D. (2000). The common C-terminal sequences of substance P and neurokinin A contact the same region of the NK-1 receptor. FEBS Lett..

[55] Davoudmanesh S., Mosaabadi J.M. (2018). Investigation of the effect of homocysteinylation of substance P on its binding to the NK1 receptor using molecular dynamics simulation. J. Mol. Model..

[56] Singh D., Joshi D.D., Hameed M., Qian J., Gascón P., Maloof P.B., Mosenthal A., Rameshwar P. (2000). Increased expression of preprotachykinin-I and neurokinin receptors in human breast cancer cells: Implications for bone marrow metastasis. Proc. Natl. Acad. Sci. USA.

[57] Davoodian M., Boroumand N., Mehrabi Bahar M., Jafarian A.H., Asadi M., Hashemy S.I. (2019). Evaluation of serum level of substance P and tissue distribution of NK-1 receptor in breast cancer. Mol. Biol. Rep..

[58] Hennig I.M., Laissue J.A., Horisberger U., Reubi J.C. (1995). Substance-P receptors in human primary neoplasms: Tumoral and vascular localization. Int. J. Cancer.

[59] Gharaee N., Pourali L., Jafarian A.H., Hashemy S.I. (2018). Evaluation of serum level of substance P and tissue distribution of NK-1 receptor in endometrial cancer. Mol. Biol. Rep..

[60] Muñoz M., Rosso M., Robles-Frias M.J., Salinas-Martín M.V., Rosso R., González-Ortega A., Coveñas R. (2010). The NK-1 receptor is expressed in human melanoma and is involved in the antitumor action of the NK-1 receptor antagonist aprepitant on melanoma cell lines. Lab. Investig..

[61] Castro T.A., Cohen M.C., Rameshwar P. (2005). The expression of neurokinin-1 and preprotachykinin-1 in breast cancer cells depends on the relative degree of invasive and metastatic potential. Clin. Exp. Metastasis.

[62] Al-Keilani M.S., Elstaty R.I., Alqudah M.A., Alkhateeb A.M. (2021). Immunohistochemical expression of substance P in breast cancer and its association with prognostic parameters and Ki-67 index. PLoS ONE.

[63] Ge C., Huang H., Huang F., Yang T., Zhang T., Wu H., Zhou H., Chen Q., Shi Y., Sun Y. (2019). Neurokinin-1 receptor is an effective target for treating leukemia by inducing oxidative stress through mitochondrial calcium overload. Proc. Natl. Acad. Sci. USA.

[64] Akazawa T., Kwatra S.G., Goldsmith L.E., Richardson M.D., Cox E.A., Sampson J.H., Kwatra M.M. (2009). A constitutively active form of neurokinin 1 receptor and neurokinin 1 receptor-mediated apoptosis in glioblastomas. J. Neurochem..

[65] Muñoz M.F., Argüelles S., Rosso M., Medina R., Coveñas R., Ayala A., Muñoz M. (2022). The Neurokinin-1 Receptor Is Essential for the Viability of Human Glioma Cells: A Possible Target for Treating Glioblastoma. BioMed Res. Int..

[66] Harford-Wright E., Lewis K.M., Vink R., Ghabriel M.N. (2014). Evaluating the role of substance P in the growth of brain tumors. Neuroscience.

[67] Zhou Y., Wang M., Tong Y., Liu X., Zhang L., Dong D., Shao J., Zhou Y. (2019). miR-206 Promotes Cancer Progression by Targeting Full-Length Neurokinin-1 Receptor in Breast Cancer. Technol. Cancer Res. Treat..

[68] Wang F., Liu S., Liu J., Feng F., Guo Y., Zhang W., Zheng G., Wang Q., Cai L., Guo M. (2019). SP promotes cell proliferation in esophageal squamous cell carcinoma through the NK1R/Hes1 axis. Biochem. Biophys. Res. Commun..

[69] Dong J., Feng F., Xu G., Zhang H., Hong L., Yang J. (2015). Elevated SP/NK-1R in esophageal carcinoma promotes esophageal carcinoma cell proliferation and migration. Gene.

[70] Deng X., Tang S., Wu P., Li Q., Ge X., Xu B., Wang H., Miao L. (2019). SP/NK-1R promotes gallbladder cancer cell proliferation and migration. J. Cell Mol. Med..

[71] Gutierrez S., Boada M.D. (2018). Neuropeptide-induced modulation of carcinogenesis in a metastatic breast cancer cell line (MDA-MB-231(LUC+)). Cancer Cell Int..

[72] Garcia-Recio S., Fuster G., Fernandez-Nogueira P., Pastor-Arroyo E.M., Park S.Y., Mayordomo C., Ametller E., Mancino M., Gonzalez-Farre X., Russnes H.G. (2013). Substance P autocrine signaling contributes to persistent HER2 activation that drives malignant progression and drug resistance in breast cancer. Cancer Res..

[73] Ziche M., Morbidelli L., Pacini M., Geppetti P., Alessandri G., Maggi C.A. (1990). Substance P stimulates neovascularization in vivo and proliferation of cultured endothelial cells. Microvasc. Res..

[74] Zhou Y., Zhao L., Xiong T., Chen X., Zhang Y., Yu M., Yang J., Yao Z. (2013). Roles of full-length and truncated neurokinin-1 receptors on tumor progression and distant metastasis in human breast cancer. Breast Cancer Res. Treat..

[75] Garcia-Recio S., Gascón P. (2015). Biological and Pharmacological Aspects of the NK1-Receptor. BioMed Res. Int..

[76] Spitsin S., Pappa V., Douglas S.D. (2018). Truncation of neurokinin-1 receptor-Negative regulation of substance P signaling. J. Leukoc. Biol..

[77] Feng F., Yang J., Tong L., Yuan S., Tian Y., Hong L., Wang W., Zhang H. (2011). Substance P immunoreactive nerve fibres are related to gastric cancer differentiation status and could promote proliferation and migration of gastric cancer cells. Cell Biol. Int..

[78] O’Connor T.M., O’Connell J., O’Brien D.I., Goode T., Bredin C.P., Shanahan F. (2004). The role of substance P in inflammatory disease. J. Cell Physiol..

[79] Muñoz M., Rosso M., Carranza A., Coveñas R. (2017). Increased nuclear localization of substance P in human gastric tumor cells. Acta Histochem..

[80] Xu M., Seas A., Kiyani M., Ji K.S.Y., Bell H.N. (2018). A temporal examination of calcium signaling in cancer- from tumorigenesis, to immune evasion, and metastasis. Cell Biosci..

[81] Muñoz M., Coveñas R. (2013). Safety of neurokinin-1 receptor antagonists. Expert. Opin. Drug Saf..

[82] Muñoz M., Rosso M., Coveñas R. (2017). Neurokinin-1 receptor antagonists in lung cancer therapy. Lett. Drug Des. Discov..

[83] Mayordomo C., García-Recio S., Ametller E., Fernández-Nogueira P., Pastor-Arroyo E.M., Vinyals L., Casas I., Gascón P., Almendro V. (2012). Targeting of substance P induces cancer cell death and decreases the steady state of EGFR and Her2. J. Cell Physiol..

[84] Covenas R., Munoz M. (2022). Involvement of the Substance P/Neurokinin-1 Receptor System in Cancer. Cancers.

[85] Andrews P.L.R., Golding J.F., Sanger G.J. (2023). An assessment of the effects of neurokinin(1) receptor antagonism against nausea and vomiting: Relative efficacy, sites of action and lessons for future drug development. Br. J. Clin. Pharmacol..

[86] Rodriguez P.L., Jiang S., Fu Y., Avraham S., Avraham H.K. (2014). The proinflammatory peptide substance P promotes blood-brain barrier breaching by breast cancer cells through changes in microvascular endothelial cell tight junctions. Int. J. Cancer.

[87] Lee M., McCloskey M., Staples S. (2016). Prolonged use of Aprepitant in metastatic breast cancer and a reduction in CA153 tumour marker levels. Int. J. Cancer Clin. Res..

[88] Muñoz M., Berger M., Rosso M., Gonzalez-Ortega A., Carranza A., Coveñas R. (2014). Antitumor activity of neurokinin-1 receptor antagonists in MG-63 human osteosarcoma xenografts. Int. J. Oncol..

[89] Mozafari M., Ebrahimi S., Darban R.A., Hashemy S.I. (2022). Potential in vitro therapeutic effects of targeting SP/NK1R system in cervical cancer. Mol. Biol. Rep..

[90] Kolorz J., Demir S., Gottschlich A., Beirith I., Ilmer M., Lüthy D., Walz C., Dorostkar M.M., Magg T., Hauck F. (2021). The Neurokinin-1 Receptor Is a Target in Pediatric Rhabdoid Tumors. Curr. Oncol..

[91] Alsaeed M.A., Ebrahimi S., Alalikhan A., Hashemi S.F., Hashemy S.I. (2022). The Potential In Vitro Inhibitory Effects of Neurokinin-1 Receptor (NK-1R) Antagonist, Aprepitant, in Osteosarcoma Cell Migration and Metastasis. BioMed Res. Int..

[92] Momen Razmgah M., Ghahremanloo A., Javid H., AlAlikhan A., Afshari A., Hashemy S.I. (2022). The effect of substance P and its specific antagonist (aprepitant) on the expression of MMP-2, MMP-9, VEGF, and VEGFR in ovarian cancer cells. Mol. Biol. Rep..

[93] Ebrahimi S., Mirzavi F., Aghaee-Bakhtiari S.H., Hashemy S.I. (2022). SP/NK1R system regulates carcinogenesis in prostate cancer: Shedding light on the antitumoral function of aprepitant. Biochim. Biophys. Acta Mol. Cell Res..

[94] Zhang X., Li L., Hu W., Hu M., Tao Y., Hu H., Miao X., Yang W., Zhu Q., Mou L. (2022). Neurokinin-1 receptor promotes non-small cell lung cancer progression through transactivation of EGFR. Cell Death Dis..

[95] Javid H., Afshari A.R., Zahedi Avval F., Asadi J., Hashemy S.I. (2021). Aprepitant Promotes Caspase-Dependent Apoptotic Cell Death and G2/M Arrest through PI3K/Akt/NF-κB Axis in Cancer Stem-Like Esophageal Squamous Cell Carcinoma Spheres. BioMed Res. Int..

[96] Shi Y., Wang X., Meng Y., Ma J., Zhang Q., Shao G., Wang L., Cheng X., Hong X., Wang Y. (2021). A Novel Mechanism of Endoplasmic Reticulum Stress- and c-Myc-Degradation-Mediated Therapeutic Benefits of Antineurokinin-1 Receptor Drugs in Colorectal Cancer. Adv. Sci..

[97] Ghahremanloo A., Javid H., Afshari A.R., Hashemy S.I. (2021). Investigation of the Role of Neurokinin-1 Receptor Inhibition Using Aprepitant in the Apoptotic Cell Death through PI3K/Akt/NF-κB Signal Transduction Pathways in Colon Cancer Cells. BioMed Res. Int..

[98] Singh S., Kumaravel S., Dhole S., Roy S., Pavan V., Chakraborty S. (2021). Neuropeptide Substance P Enhances Inflammation-Mediated Tumor Signaling Pathways and Migration and Proliferation of Head and Neck Cancers. Indian J. Surg. Oncol..

[99] Mohammadi F., Javid H., Afshari A.R., Mashkani B., Hashemy S.I. (2020). Substance P accelerates the progression of human esophageal squamous cell carcinoma via MMP-2, MMP-9, VEGF-A, and VEGFR1 overexpression. Mol. Biol. Rep..

[100] Matalińska J., Świć A., Lipiński P., Misicka A. (2020). Antiproliferative effects of [D-Pro2, D-Trp7,9]-Substance P and aprepitant on several cancer cell lines and their selectivity in comparison to normal cells. Folia Neuropathol..

[101] Bayati S., Razani E., Bashash D., Safaroghli-Azar A., Safa M., Ghaffari S.H. (2018). Antileukemic effects of neurokinin-1 receptor inhibition on hematologic malignant cells: A novel therapeutic potential for aprepitant. Anticancer Drugs.

[102] Zhang L., Wang L., Dong D., Wang Z., Ji W., Yu M., Zhang F., Niu R., Zhou Y. (2019). MiR-34b/c-5p and the neurokinin-1 receptor regulate breast cancer cell proliferation and apoptosis. Cell Prolif..

[103] Nizam E., Erin N. (2018). Differential consequences of neurokinin receptor 1 and 2 antagonists in metastatic breast carcinoma cells; Effects independent of Substance P. Biomed. Pharmacother..

[104] Dikmen M., Gökhaner G., Cantürk Z. (2019). Evaluation of the antileukemic effects of neurokinin-1 receptor antagonists, aprepitant, and L-733,060, in chronic and acute myeloid leukemic cells. Anticancer Drugs.

[105] Wu H., Cheng X., Huang F., Shao G., Meng Y., Wang L., Wang T., Jia X., Yang T., Wang X. (2020). Aprepitant Sensitizes Acute Myeloid Leukemia Cells to the Cytotoxic Effects of Cytosine Arabinoside in vitro and in vivo. Drug Des. Devel Ther..

[106] Nowicki M., Ostalska-Nowicka D., Kondraciuk B., Miskowiak B. (2007). The significance of substance P in physiological and malignant haematopoiesis. J. Clin. Pathol..

[107] Nizam E., Köksoy S., Erin N. (2020). NK1R antagonist decreases inflammation and metastasis of breast carcinoma cells metastasized to liver but not to brain; phenotype-dependent therapeutic and toxic consequences. Cancer Immunol. Immunother..

[108] Guan L., Yuan S., Ma J., Liu H., Huang L., Zhang F. (2023). Neurokinin-1 receptor is highly expressed in cervical cancer and its antagonist induces cervical cancer cell apoptosis. Eur. J. Histochem..

[109] Meng F., DeMorrow S., Venter J., Frampton G., Han Y., Francis H., Standeford H., Avila S., McDaniel K., McMillin M. (2014). Overexpression of membrane metalloendopeptidase inhibits substance P stimulation of cholangiocarcinoma growth. Am. J. Physiol. Gastrointest. Liver Physiol..

[110] Lorestani S., Ghahremanloo A., Jangjoo A., Abedi M., Hashemy S.I. (2020). Evaluation of serum level of substance P and tissue distribution of NK-1 receptor in colorectal cancer. Mol. Biol. Rep..

[111] Golestaneh M., Firoozrai M., Javid H., Hashemy S.I. (2022). The substance P/ neurokinin-1 receptor signaling pathway mediates metastasis in human colorectal SW480 cancer cells. Mol. Biol. Rep..

[112] Javid H., Asadi J., Zahedi Avval F., Afshari A.R., Hashemy S.I. (2020). The role of substance P/neurokinin 1 receptor in the pathogenesis of esophageal squamous cell carcinoma through constitutively active PI3K/Akt/NF-κB signal transduction pathways. Mol. Biol. Rep..

[113] Afshari A.R., Motamed-Sanaye A., Sabri H., Soltani A., Karkon-Shayan S., Radvar S., Javid H., Mollazadeh H., Sathyapalan T., Sahebkar A. (2021). Neurokinin-1 Receptor (NK-1R) Antagonists: Potential Targets in the Treatment of Glioblastoma Multiforme. Curr. Med. Chem..

[114] Rezaei S., Assaran Darban R., Javid H., Hashemy S.I. (2022). The Therapeutic Potential of Aprepitant in Glioblastoma Cancer Cells through Redox Modification. BioMed Res. Int..

[115] Muñoz M., Rosso M., Coveñas R. (2019). Neurokinin-1 Receptor Antagonists against Hepatoblastoma. Cancers.

[116] Hongfeng Z., Andong J., Liwen S., Mingping B., Xiaowei Y., Mingyong L., Aimin Y. (2020). lncRNA RMRP knockdown suppress hepatocellular carcinoma biological activities via regulation miRNA-206/TACR1. J. Cell Biochem..

[117] Ilmer M., Garnier A., Vykoukal J., Alt E., von Schweinitz D., Kappler R., Berger M. (2015). Targeting the Neurokinin-1 Receptor Compromises Canonical Wnt Signaling in Hepatoblastoma. Mol. Cancer Ther..

[118] Esteban F., Ramos-García P., Muñoz M., González-Moles M.Á. (2021). Substance P and Neurokinin 1 Receptor in Chronic Inflammation and Cancer of the Head and Neck: A Review of the Literature. Int. J. Environ. Res. Public. Health.

[119] Borrego J.F., Huelsmeyer M.K., Pinkerton M.E., Muszynski J.L., Miller S.A.K., Kurzman I.D., Vail D.M. (2016). Neurokinin-1 receptor expression and antagonism by the NK-1R antagonist maropitant in canine melanoma cell lines and primary tumour tissues. Vet. Comp. Oncol..

[120] Zhang X., Li J., Li L., Hu W., Tao Y., Gao W., Ye Z., Jia H., Wang J., Miao X. (2023). Neurokinin-1 receptor drives PKCɑ-AURKA/N-Myc signaling to facilitate the neuroendocrine progression of prostate cancer. Cell Death Dis..

[121] Zhou J., Geng K., Ping F., Gao Y., Liu L., Feng B. (2016). Cross-talk between 5-hydroxytryptamine and substance P in the melanogensis and apoptosis of B16F10 melanoma cells. Eur. J. Pharmacol..

[122] Berger M., VON Schweinitz D. (2017). Therapeutic Innovations for Targeting Childhood Neuroblastoma: Implications of the Neurokinin-1 Receptor System. Anticancer Res..

[123] Henssen A.G., Odersky A., Szymansky A., Seiler M., Althoff K., Beckers A., Speleman F., Schäfers S., De Preter K., Astrahanseff K. (2017). Targeting tachykinin receptors in neuroblastoma. Oncotarget.

[124] Pohl A., Kappler R., Mühling J., VON Schweinitz D., Berger M. (2017). Expression of Truncated Neurokinin-1 Receptor in Childhood Neuroblastoma is Independent of Tumor Biology and Stage. Anticancer Res..

[125] Pan B., Liu D., Yang L., Wüthrich K. (2022). GPCR large-amplitude dynamics by (19)F-NMR of aprepitant bound to the neurokinin 1 receptor. Proc. Natl. Acad. Sci. USA.

[126] AlAlikhan A., Ghahremanloo A., Javid H., Ebrahimi S., Hashemy S.I. (2022). The Effect of Blocking Neurokinin-1 Receptor by Aprepitant on the Inflammatory and Apoptosis Pathways in Human Ovarian Cancer Cells. Cell Biochem. Biophys..

[127] Huang C., Li Y., Guo Y., Zhang Z., Lian G., Chen Y., Li J., Su Y., Li J., Yang K. (2018). MMP1/PAR1/SP/NK1R paracrine loop modulates early perineural invasion of pancreatic cancer cells. Theranostics.

[128] Ji T., Ma K., Wu H., Cao T. (2022). A Substance P (SP)/Neurokinin-1 Receptor Axis Promotes Perineural Invasion of Pancreatic Cancer and Is Affected by lncRNA LOC389641. J. Immunol. Res..

[129] González-Santana A., Marrero-Hernández S., Dorta I., Hernández M., Pinto F.M., Báez D., Bello A.R., Candenas L., Almeida T.A. (2016). Altered expression of the tachykinins substance P/neurokinin A/hemokinin-1 and their preferred neurokinin 1/neurokinin 2 receptors in uterine leiomyomata. Fertil. Steril..

[130] Fackler O.T., Grosse R. (2008). Cell motility through plasma membrane blebbing. J. Cell Biol..

[131] Meshki J., Douglas S.D., Lai J., Schwartz L., Kilpatrick L.E., Tuluc F. (2009). Neurokinin 1 receptor mediates membrane blebbing in HEK293 cells through a Rho/Rho-associated coiled-coil kinase-dependent mechanism. J. Biol. Chem..

[132] Coveñas R., Rodríguez F.D., Muñoz M. (2022). The Neurokinin-1 Receptor: A Promising Antitumor Target. Receptors.

[133] Niu X., Hou J., Li J. (2018). The NK1 receptor antagonist NKP608 inhibits proliferation of human colorectal cancer cells via Wnt signaling pathway. Biol. Res..

[134] Zhou J., Ling J., Song H., Lv B., Wang L., Shang J., Wang Y., Chang C., Ping F., Qian J. (2016). Neurokinin-1 receptor is a novel positive regulator of Wnt/ β-catenin signaling in melanogenesis. Oncotarget.

[135] Godwin P., Baird A.M., Heavey S., Barr M.P., O’Byrne K.J., Gately K. (2013). Targeting nuclear factor-kappa B to overcome resistance to chemotherapy. Front. Oncol..

[136] Ständer S., Siepmann D., Herrgott I., Sunderkötter C., Luger T.A. (2010). Targeting the neurokinin receptor 1 with aprepitant: A novel antipruritic strategy. PLoS ONE.

[137] Roila F., Rolski J., Ramlau R., Dediu M., Russo M.W., Bandekar R.R., Grunberg S.M. (2009). Randomized, double-blind, dose-ranging trial of the oral neurokinin-1 receptor antagonist casopitant mesylate for the prevention of cisplatin-induced nausea and vomiting. Ann. Oncol..

[138] Smith J.A., Harle A., Dockry R., Holt K., Russell P., Molassiotis A., Yorke J., Robinson R., Birrell M.A., Belvisi M.G. (2021). Aprepitant for Cough in Lung Cancer. A Randomized Placebo-controlled Trial and Mechanistic Insights. Am. J. Respir. Crit. Care Med..

[139] Noronha V., Bhattacharjee A., Patil V.M., Joshi A., Menon N., Shah S., Kannan S., Mukadam S.A., Maske K., Ishi S. (2020). Aprepitant for Cough Suppression in Advanced Lung Cancer: A Randomized Trial. Chest.

[140] Naito T., Suzuki Y., Shibata K., Kawakami J. (2021). Simple Liquid Chromatography-Tandem Mass Spectrometry Method for Quantitation of Total and Free Aprepitant and Its Active N-Dealkylated Metabolites in Human Plasma. Ther. Drug Monit..

[141] Muñoz M., Coveñas R. (2019). Neurokinin-1 Receptor Antagonists as Anticancer Drugs. Lett. Drug Des. Discov..

[142] Garnier A., Ilmer M., Kappler R., Berger M. (2016). Therapeutic Innovations for Targeting Hepatoblastoma. Anticancer Res..

[143] Wang J., Yu J., Hu J., Yang W., Ren H., Ding D., Zhang L., Liu X. (2015). Neurokinin-1 activation affects EGFR related signal transduction in triple negative breast cancer. Cell Signal.

[144] Robinson P., Kasembeli M., Bharadwaj U., Engineer N., Eckols K.T., Tweardy D.J. (2016). Substance P Receptor Signaling Mediates Doxorubicin-Induced Cardiomyocyte Apoptosis and Triple-Negative Breast Cancer Chemoresistance. BioMed Res. Int..

[145] Eblen S.T., Slack J.K., Weber M.J., Catling A.D. (2002). Rac-PAK signaling stimulates extracellular signal-regulated kinase (ERK) activation by regulating formation of MEK1-ERK complexes. Mol. Cell Biol..

[146] Al-Keilani M., Bdeir R., Elstaty R.I., Alqudah M.A. (2023). Expression of substance P, neurokinin 1 receptor, Ki-67 and pyruvate kinase M2 in hormone receptor negative breast cancer and evaluation of impact on overall survival. BMC Cancer.

[147] Kitchens C.A., McDonald P.R., Pollack I.F., Wipf P., Lazo J.S. (2009). Synergy between microtubule destabilizing agents and neurokinin 1 receptor antagonists identified by an siRNA synthetic lethal screen. FASEB J..

[148] Alfieri A.B., Cubeddu L.X. (2004). Efectos de los antagonistas de los receptores NK1 y de la dexametasona sobre la inflamación neurogénica inducida por ciclofosfamida y por radiación X, en la rata. Arch. Venez. Farmacol. Ter..

[149] Alfieri A.B., Cubeddu L.X. (2000). Role of NK1 receptors on cisplatin-induced nephrotoxicity in the rat. Naunyn Schmiedebergs Arch. Pharmacol..

[150] Molinos-Quintana A., Trujillo-Hacha P., Piruat J.I., Bejarano-García J.A., García-Guerrero E., Pérez-Simón J.A., Muñoz M. (2019). Human acute myeloid leukemia cells express Neurokinin-1 receptor, which is involved in the antileukemic effect of Neurokinin-1 receptor antagonists. Investig. New Drugs.

[151] Muñoz M., Crespo J.C., Crespo J.P., Coveñas R. (2019). Neurokinin-1 receptor antagonist aprepitant and radiotherapy, a successful combination therapy in a patient with lung cancer: A case report. Mol. Clin. Oncol..

[152] Berger M., Neth O., Ilmer M., Garnier A., Salinas-Martin M.V., de Agustin Asencio J.C., von Schweinitz D., Kappler R., Munoz M. (2014). Hepatoblastoma cells express truncated neurokinin-1 receptor and can be growth inhibited by aprepitant in vitro and in vivo. J. Hepatol..

[153] Rupniak N.M.J., Kramer M.S. (2017). NK1 receptor antagonists for depression: Why a validated concept was abandoned. J. Affect. Disord..

[154] Ratti E., Bettica P., Alexander R., Archer G., Carpenter D., Evoniuk G., Gomeni R., Lawson E., Lopez M., Millns H. (2013). Full central neurokinin-1 receptor blockade is required for efficacy in depression: Evidence from orvepitant clinical studies. J. Psychopharmacol..

[155] Okumura L.M., da Silva Ries S.A., Meneses C.F., Michalowski M.B., Ferreira M.A.P., Moreira L.B. (2019). Adverse events associated with aprepitant pediatric bone cancer patients. J. Oncol. Pharm. Pract..

[156] Bukowski K., Kciuk M., Kontek R. (2020). Mechanisms of Multidrug Resistance in Cancer Chemotherapy. Int. J. Mol. Sci..

[157] Bashash D., Safaroghli-Azar A., Bayati S., Razani E., Pourbagheri-Sigaroodi A., Gharehbaghian A., Momeny M., Sanjadi M., Rezaie-Tavirani M., Ghaffari S.H. (2018). Neurokinin-1 receptor (NK1R) inhibition sensitizes APL cells to anti-tumor effect of arsenic trioxide via restriction of NF-κB axis: Shedding new light on resistance to Aprepitant. Int. J. Biochem. Cell Biol..

[158] Ebrahimi S., Mirzavi F., Hashemy S.I., Khaleghi Ghadiri M., Stummer W., Gorji A. (2023). The in vitro anti-cancer synergy of neurokinin-1 receptor antagonist, aprepitant, and 5-aminolevulinic acid in glioblastoma. Biofactors.

[159] Cao X., Yang Y., Zhou W., Wang Y., Wang X., Ge X., Wang F., Zhou F., Deng X., Miao L. (2023). Aprepitant inhibits the development and metastasis of gallbladder cancer via ROS and MAPK activation. BMC Cancer.

[160] Zheng Y., Sang M., Liu F., Gu L., Li J., Wu Y., Shan B. (2023). Aprepitant inhibits the progression of esophageal squamous cancer by blocking the truncated neurokinin-1 receptor. Oncol. Rep..

[161] Zhao M., Wang T., Liu Q., Cummins S. (2017). Copy number alteration of neuropeptides and receptors in multiple cancers. Sci. Rep..

[162] Rapoport B.L., Jordan K., Weinstein C. (2018). Neurokinin 1 receptor antagonists in the prevention of chemotherapy-induced nausea and vomiting: Focus on fosaprepitant. Future Oncol..

[163] Navari R.M. (2008). Fosaprepitant: A neurokinin-1 receptor antagonist for the prevention of chemotherapy-induced nausea and vomiting. Expert. Rev. Anticancer. Ther..

[164] Kanduluru A.K., Srinivasarao M., Wayua C., Low P.S. (2020). Evaluation of a Neurokinin-1 Receptor-Targeted Technetium-99m Conjugate for Neuroendocrine Cancer Imaging. Mol. Imaging Biol..

[165] Kanduluru A.K., Low P.S. (2017). Development of a Ligand-Targeted Therapeutic Agent for Neurokinin-1 Receptor Expressing Cancers. Mol. Pharm..

[166] Recio R., Vengut-Climent E., Mouillac B., Orcel H., López-Lázaro M., Calderón-Montaño J.M., Álvarez E., Khiar N., Fernández I. (2017). Design, synthesis and biological studies of a library of NK1-Receptor Ligands Based on a 5-arylthiosubstituted 2-amino-4,6-diaryl-3-cyano-4H-pyran core: Switch from antagonist to agonist effect by chemical modification. Eur. J. Med. Chem..

[167] Recio R., Lerena P., Pozo E., Calderón-Montaño J.M., Burgos-Morón E., López-Lázaro M., Valdivia V., Pernia Leal M., Mouillac B., Organero J.Á. (2021). Carbohydrate-Based NK1R Antagonists with Broad-Spectrum Anticancer Activity. J. Med. Chem..

[168] Efficacy and Safety of PVT-1 Treatment in Patients with Advanced Non-Small Cell Lung Cancer. https://www.clinicaltrials.gov/study/NCT04840004.

